# Skin-picking disorder and trichotillomania in Down syndrome: A TriNetX-based cohort study

**DOI:** 10.1016/j.jdin.2026.09.012

**Published:** 2026-10-05

**Authors:** Nuran C. Golbasi, Shari R. Lipner

**Affiliations:** a Weill Cornell Medicine, New York, New York; b Department of Dermatology, Weill Cornell Medicine, New York, New York

**Keywords:** body-focused repetitive behaviors, Cox proportional hazards model, Down syndrome, epidemiology, neurodevelopmental disorders, psychiatric comorbidity, retrospective cohort, skin-picking disorder, trichotillomania, TriNetX

*To the Editor:* Down syndrome (DS) is a genetic neurodevelopmental condition associated with psychiatric and behavioral comorbidities. Prior studies suggest increased repetitive and compulsive-like behaviors in individuals with DS. However, the longitudinal association between body-focused repetitive behaviors (BFRBs) and DS is uncharacterized.^1^, ^2^, ^3^, ^4^ Therefore, we evaluated this relationship using TriNetX, a multicenter database of ≥100 health care organizations with internal data validation.

On 8/12/2026, TriNetX was queried for individuals with first recorded diagnosis of DS versus controls without DS, 2005 to 2025. Baseline patient encounter was required; prior BFRBs were excluded. Cohorts were propensity matched on demographics, comorbidities, and health care utilization (Supplementary Table I, available via Mendeley at https://data.mendeley.com/datasets/bcjrm6gwnf/1). Five-year incidence proportions and risk differences (RDs) were calculated. Hazard ratios (HRs) were estimated over 5 years using Cox proportional hazards models, with proportionality assessed using a Schoenfeld residual–based test. Bonferroni correction was applied for 2 prespecified outcomes, with *P* < .025 considered significant.

After matching, baseline characteristics were well balanced (Table I). Over 5 years, DS versus controls showed higher incidence of skin-picking disorder (0.12% vs 0.06%; HR = 1.89; 95% confidence interval [CI]: 1.29-2.75; RD = 0.06% [95% CI: 0.02-0.09]) and trichotillomania (0.35% vs 0.05%; HR = 7.73 [95% CI: 5.32-11.24]; RD = 0.31% [95% CI: 0.26-0.35]), with low absolute event rates (Table II). Age-stratified analyses showed elevated trichotillomania hazards in children (aged ≤17 years) and adults (aged ≥18 years) and elevated skin-picking hazard only in adults. Associations remained elevated in sensitivity analyses requiring ≥2 DS diagnoses, using a congenital cleft lip/palate comparator, and matching on neurodevelopmental covariates (Supplementary Table II, available via Mendeley at https://data.mendeley.com/datasets/bcjrm6gwnf/1).

**Table I:** Baseline characteristics of patients with Down syndrome and matched controls

Characteristic	DS (*N* = 68,243)	Controls (*N* = 68,243)	SMD
Demographics			
Age, y	26.3 ± 19.3	26.3 ± 19.3	0.001
Female sex	33,703 (49.4%)	33,709 (49.4%)	<0.001
White race	45,432 (66.6%)	45,423 (66.6%)	<0.001
Black or African American race	8052 (11.8%)	8057 (11.8%)	<0.001
Asian race	2410 (3.5%)	2410 (3.5%)	<0.001
Hispanic ethnicity	13,616 (20.0%)	13,609 (19.9%)	<0.001
Comorbidities			
Anxiety	1388 (2.0%)	1380 (2.0%)	0.001
Depression	1136 (1.7%)	1124 (1.6%)	0.001
Atopic dermatitis	360 (0.5%)	356 (0.5%)	0.001
Pruritus	284 (0.4%)	284 (0.4%)	<0.001
Health care utilization			
≥1 outpatient office visit	16,509 (24.2%)	16,507 (24.2%)	<0.001

Categorical variables are reported as *n* (%) and continuous variables are reported as mean ± standard deviation (SD). Postmatch standardized mean differences (SMDs) <0.10 indicate adequate covariate balance. Median follow-up was 1789 days (IQR, 1318) in the DS cohort and 1532 days (IQR, 1271) in controls. Conditions were identified using ICD-10 codes Q90 (Down syndrome [DS]), F41 (anxiety), F32 (depression), L20 (atopic dermatitis), and L29 (pruritus); full code definitions are provided in Supplementary Table I, available via Mendeley at https://data.mendeley.com/datasets/bcjrm6gwnf/1.

*DS*, Down syndrome; *SMD*, standardized mean difference.

**Table II:** Five-year incidence proportions and hazard ratios of body-focused repetitive behaviors in individuals with Down syndrome versus matched controls

Outcome	DS, *n* (%)	Controls, *n* (%)	RD, % (95% CI)	HR (95% CI)	*P* value
Skin-picking disorder	79 (0.12%)	41 (0.06%)	0.06 (0.02-0.09)	1.89 (1.29-2.75)	.001∗
Trichotillomania	240 (0.35%)	31 (0.05%)	0.31 (0.26-0.35)	7.73 (5.32-11.24)	<.001∗

Percentages represent 5-year incidence proportions following matching (*N* = 68,243 per cohort). Risk differences (RDs) are expressed in percentage points. Cox proportional hazards models estimated hazard ratios (HRs) with 95% confidence intervals (CIs); no violations of the proportional hazards assumption were observed. Outcomes were defined using ICD-10 codes F42.4 (skin-picking disorder) and F63.3 (trichotillomania); full code definitions are provided in Supplementary Table I, available via Mendeley at https://data.mendeley.com/datasets/bcjrm6gwnf/1.

*CI*, Confidence interval; *DS*, Down syndrome; *HR*, hazard ratio; *RD*, risk difference.

∗ *P* values are shown unadjusted with statistical significance defined as *P* < .025 after Bonferroni correction.

We found that DS was associated with increased hazard of trichotillomania and skin-picking disorder over 5 years, consistent with prior literature. In a 2-year longitudinal cohort (*n* = 85), repetitive behaviors were more frequent and severe in DS (*P* < .01) and decreased with age only in controls.^1^ In a cross-sectional cohort of patients with DS (*n* = 206), routinized and compulsive-like behaviors did not decline with increasing developmental levels.^2^ In another cross-sectional cohort of children with DS (*n* = 100), trichotillomania was observed in 4%.^3^

BFRBs in individuals with DS may be under-recognized in clinical practice, and reliance on diagnostic coding likely underestimates their clinical burden. A patient-perspective report highlighted skin-picking as under-recognized in DS and emphasized the importance of patient-centered screening.^4^ Many affected individuals do not seek treatment, and symptoms may persist for years if unrecognized, potentially leading to tissue damage, scarring, alopecia, infection, and psychosocial impairment.^5^

Mechanistically, self-regulatory and anxiety-related behaviors in DS may increase vulnerability to BFRBs, which can be reinforced by distress relief.^1^, ^2^, ^5^ Recognition should prompt assessment of behavioral triggers and psychiatric comorbidities to inform management.

Limitations include residual confounding, reliance on diagnostic coding, differential loss to follow-up, and competing mortality. Individuals with DS may have greater health care contact and differing patterns of specialty care, which can contribute to ascertainment bias. Low event counts may limit stability of HR estimates, particularly for skin-picking disorder. Other BFRBs lacked specific International Classification of Diseases, 10th Revision codes. Association does not imply causation.

In sum, DS was associated with increased hazard of skin-picking and trichotillomania. Increased clinical awareness of BFRBs in DS may facilitate recognition and management. Prospective studies with severity assessments could characterize clinical impact.

### Declaration of generative AI and AI-assisted technologies in the writing process

AI was not used in the preparation of this work.

## Conflicts of interest

Dr Lipner has served as consultant for Moberg Pharmaceuticals, Veradermics, and BelleTorus Corporation. Nuran C. Golbasi has no conflicts of interest to declare.

## References

[1] Evans D.W., Kleinpeter F.L., Slane M.M., Boomer K.B. (2014). Adaptive and maladaptive correlates of repetitive behavior and restricted interests in persons with Down syndrome and developmentally-matched typical children: a two-year longitudinal sequential design. PLoS One.

[2] Glenn S., Cunningham C., Nananidou A., Prasher V., Glenholmes P. (2015). Routinised and compulsive-like behaviours in individuals with Down syndrome. J Intellect Disabil Res.

[3] Daneshpazhooh M., Nazemi T.M., Bigdeloo L., Yoosefi M. (2007). Mucocutaneous findings in 100 children with Down syndrome. Pediatr Dermatol.

[4] Epstein Z., Epstein L., Rork J., Koo J., Kranyak A. (2025). Skin picking in Down syndrome: a patient speaks out. Br J Dermatol.

[5] Grant J.E., Chamberlain S.R. (2021). Trichotillomania and skin-picking disorder: an update. Focus (Am Psychiatr Publ).

